# Predicting the pathological invasiveness of early lung adenocarcinoma prior to surgery using Deauville criteria: reliability and validity

**DOI:** 10.1007/s11604-023-01397-z

**Published:** 2023-02-08

**Authors:** Miki Nishimori, Hitomi Iwasa, Kosuke Nakaji, Noriko Nitta, Kana Miyatake, Rika Yoshimatsu, Tomoaki Yamanishi, Tomohiro Matsumoto, Mahiru Kato, Naoya Hayashi, Makoto Toi, Masaya Tamura, Takuji Yamagami

**Affiliations:** 1 Department of Diagnostic and Interventional Radiology, Kochi Medical School, Kochi University, Kohasu, Oko-Cho, Nankoku, Kochi 783-8505 Japan; 2 Center for Innovative and Translational Medicine, Kochi Medical School, Kochi University, Kohasu, Oko-Cho, Nankoku, Kochi 783-8505 Japan; 3 Division of Radiology, Department of Medical Technology, Kochi Medical School, Kochi University, Kohasu, Oko-Cho, Nankoku, Kochi 783-8505 Japan; 4 Department of Diagnostic Pathology, Kochi Medical School, Kochi University, Kohasu, Oko-Cho, Nankoku, Kochi 783-8505 Japan; 5 Department of Thoracic Surgery, Kochi Medical School, Kochi University, Kohasu, Oko-Cho, Nankoku, Kochi 783-8505 Japan

**Keywords:** Deauville criteria, Lung adenocarcinoma, FDG-PET/CT

## Abstract

**Purpose:**

This retrospective study aimed to investigate the validity and reliability of FDG-PET/CT visual assessment using Deauville criteria to predict pathological invasiveness of early lung adenocarcinoma prior to surgery.

**Materials and methods:**

Between April 2020 and January 2022, 51 patients who underwent surgery for pathological stage 0/I lung adenocarcinoma were enrolled. The pulmonary lesions were divided into two groups according to pathological invasiveness: less invasive (including adenocarcinoma in situ and minimally invasive adenocarcinoma and invasive adenocarcinoma. We compared CT size (total and solid size), SUVmax, and Deauville score between the two groups. Furthermore, we investigated inter-rater and intra-rater agreements regarding the Deauville score. Receiver operating characteristic (ROC) curve analysis was performed to identify the diagnostic performance of each method.

**Results:**

Based on pathologic diagnoses, 51 lesions in the 51 patients were divided into 6 less invasive and 45 invasive adenocarcinoma lesions. According to quadratic-weighted Kappa statistics, inter-rater (*k* = 0.93) and intra-rater (*k* = 0.97) agreements among all five components of the Deauville score indicated high agreement. There was a statistically significant difference in CT solid size, SUVmax, and Deauville score between the two groups. There were no significant differences between CT solid size and FDG-PET/CT assessments (AUC = 0.93 for Deauville score and SUVmax, AUC = 0.84 for CT solid size).

**Conclusion:**

FDG-PET/CT visual assessment using the Deauville score could assist in deciding upon minimally invasive surgery for early lung adenocarcinoma.

## Introduction

Lung cancer is the leading cause of cancer-related mortality globally, and lung adenocarcinoma is the most frequent histologic subtype [1]. The conventional surgical procedure for lung adenocarcinoma is lobotomy with systematic mediastinal lymphadenectomy. However, in patients with adenocarcinoma in situ (AIS) and minimally invasive adenocarcinoma (MIA) prognosis for survival is good after surgical resection, and recurrence and lymph node metastasis in these patients are rare [2–4]. Therefore, Zhang et al. reported that sublobar resection without lymph node dissection might be the preferred surgical procedure for patients with AIS/MIA [5]. Results of pathology, however, cannot be obtained before surgery. For patients with early lung adenocarcinoma forecasting pathological invasiveness before surgery would be beneficial.

Fluorodeoxyglucose positron emission tomography/computed tomography (FDG-PET/CT) is an important imaging modality for the diagnosis and staging of malignancies [6, 7]. The maximum standardized uptake value (SUVmax) is a good preoperative predictor of the invasiveness of pulmonary adenocarcinoma, similar to findings of CT [8, 9]. However, to our knowledge, no study has focused on the correlation between the visual assessment of FDG-PET/CT findings using Deauville criteria and pathological invasiveness of early lung adenocarcinoma.

This retrospective study aimed to investigate the validity and reliability of the Deauville score to predict pathological invasiveness in patients with early lung adenocarcinoma.

## Materials and methods

This research was approved by the Ethical Review Board of our institution. Because this was a retrospective study, the requirement of informed consent by participants was waived. Patients’ records and information were anonymized and de-identified before analysis.

### Patients

Study participants had pathological 0/I stage lung adenocarcinoma, had undergone surgery at our Department of Thoracic Surgery, and had preoperative FDG-PET/CT within 3 months before surgery between April 2020 and January 2022.

Exclusion criteria were no thin-slice CT data, chemotherapy just before PET/CT and surgery, previous lung cancer surgery, simultaneous lung cancer upon surgical pathological examination and blood sugar ≧ 150 mg/dL.

Atypical adenomatous hyperplasia (AAH) is typically distinguishable from invasive lung adenocarcinoma due to imaging features [10]. Furthermore, the adenocarcinoma variant subtype is considered to be a relatively rare histological subtype. For the relatively common invasive mucinous adenocarcinomas, several imaging characteristics were reported, and mucinous MIA/AIS are extremely rare [11, 12]. Thus, we excluded those subtypes, such as AAH and adenocarcinoma variant subtype, from the study.

### PET/CT Scanning

After fasting for at least 6 h and receiving an intravenous injection of FDG (3.5 MBq/kg), all patients relaxed for about 60 min. Using a PET/CT scanner (Discovery MI, GE Healthcare, Waukesha, WI, USA) with a 64-slice CT component, images from the head to the upper thigh were obtained in the three-dimensional mode for 2 min at each bed position with the patient in a supine position. Non-contrast-enhanced CT images were acquired in the helical mode. A Bayesian penalized likelihood (BPL) reconstruction approach was used to reconstruct the obtained data (Q. clear; GE Healthcare). A *β* value of 400 was applied to the BPL algorithm. Respiratory gating was performed using a data-driven gating algorithm from GE Healthcare [13]. All patients were scanned with their arms by their sides. All images obtained by CT, PET, and PET/CT were examined.

### Evaluation of FDG-PET/CT

PET/CT was evaluated by the imaging analysis software SAI viewer^®^ (Fuji Medical Systems, Tokyo, Japan). In the semiquantitative analysis, the three-dimensional volume of interest to sufficiently cover the primary lesion was set on a PET/CT image, and the SUVmax was automatically measured. The SUVmax is the maximum value of a volume of interest.

For the visual analysis, FDG accumulation was evaluated based on the following Deauville five-point scale: [14]. 1, no FDG uptake above the background; 2, FDG uptake ≤ than that of the mediastinum; 3, FDG uptake > than that of the mediastinum but ≤ than that of the liver; 4, FDG uptake moderately higher than that of liver; and 5, FDG uptake markedly stronger than that of liver.

### Thin-slice computed tomography

Using thin-slice CT (<slice thickness 2 mm) and based on lung window manifestations, the ground glass nodule (GGN) component was defined as an area of hazy increased opacity of the lung with preservation of bronchial and vascular margins, whereas the solid component was defined as a patch completely obscuring the underlying lung parenchyma. Total size of the ground glass and/or solid components was expressed by the single largest dimension measured utilizing thin sections and multiplanar reconstructions on CT.

### Pathological evaluations

Surgically resected tissues were fixed in 10% neutral buffered formalin, sectioned into 4-μm thick slices, and stained with hematoxylin and eosin. Pathological classifications were according to the criteria of the 2011 IASLC/ATS/ERS classification and the 8th edition UICC-TNM classification [15, 16]. Histological grade was classified into three categories: Grade 1 (adenocarcinoma in situ, minimally invasive carcinoma, and lepidic predominant subtype of invasive adenocarcinoma), Grade 2 (acinar and papillary), and Grade 3 (solid and micropapillary). A pathologist with more than 10 years of diagnostic expertise performed the pathological analysis.

### Investigated parameters

Patients were divided into two groups according to pathological invasiveness: less invasive (including AIS and MIA) and invasive adenocarcinoma groups.

We assessed (1) the size of total and solid areas from thin-slice CT imaging and (2) the SUVmax and Deauville score from FDG-PET/CT imaging and after the assessment classified the results as less invasive or invasive adenocarcinoma.

To assess inter-rater reliability, one radiologist with 9 years’ experience with PET (rater 1) and a medical student in her second year (rater 2) separately assessed the Deauville scores. Rater 2 had no prior specific radiological knowledge or practical experience in FDG-PET/CT image evaluation. Instruction of Rater 2 took place in less than two hours through use of a publication regarding the Deauville score [14, 17] and by instructions from rater 1. Furthermore, to assess intra-rater reliability, rater 1 re-examined the same PET imaging 3 months later blinded to the original scores.

### Statistical analysis

Statistical analysis was performed using the R software (version 4.13, http://www.r-project.org). Descriptive data were expressed as the median (range). All continuous and ordinal variables were computed using the Wilcoxon-Mann–Whitney Test (‘coin’ package). Spearman's rank correlation coefficients were used to evaluate the relationships between SUVmax and the Deauville score (‘stats’ package). For visual assessment, quadratic-weighted Kappa statistics (‘irr’ package) were used to quantify inter-rater and intra-rater reliability. Receiver operating characteristic (ROC) curve analysis was performed to identify the diagnostic performance of each method (‘pROC’ package). A *p* value of < 0.05 indicated a statistically significant difference.

## Results

### Clinical characteristics of lung adenocarcinoma

**Fig. 1:**
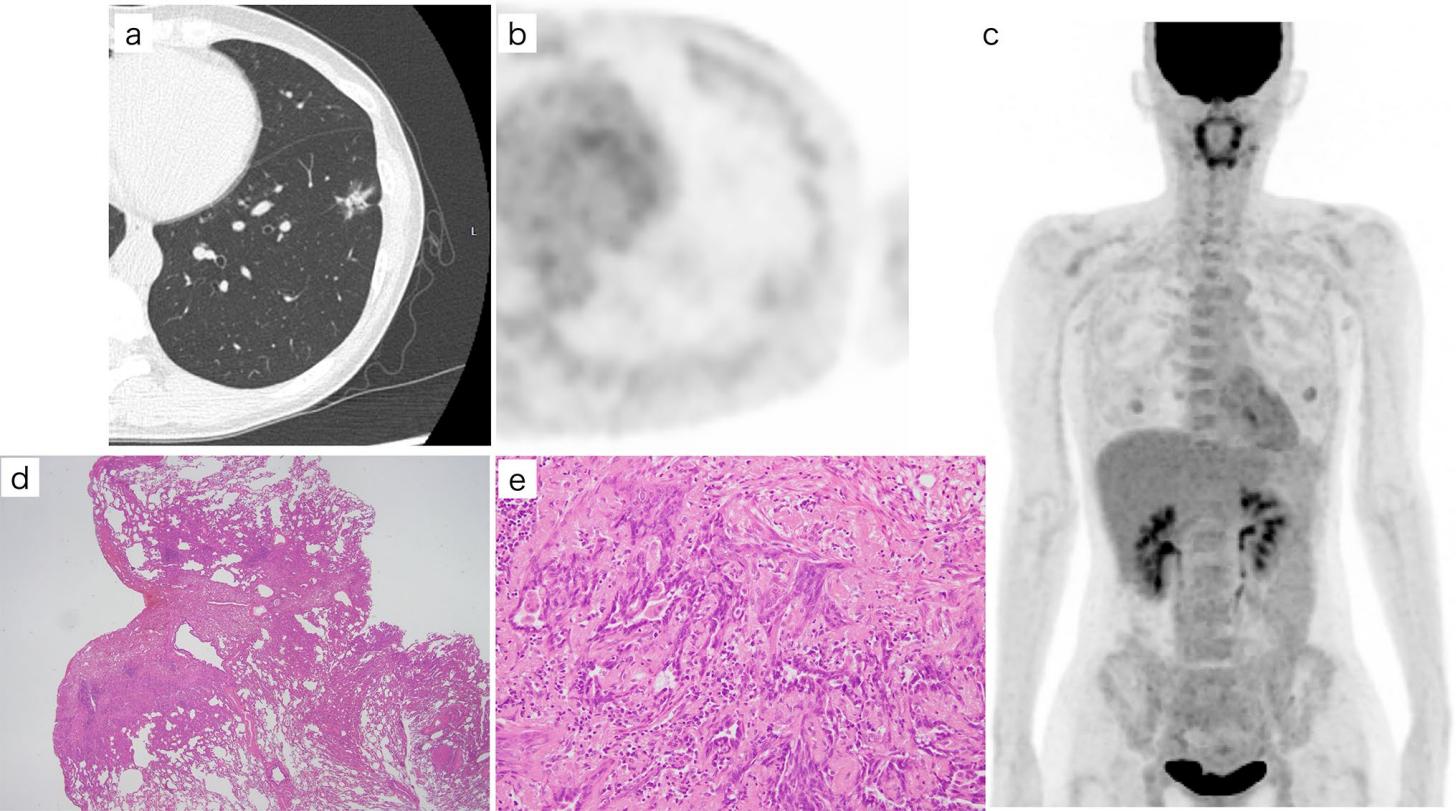
FDG-PET/CT and pathological images of a patient with minimally invasive adenocarcinoma (MIA) of the lung. **a** CT of a 46-year-old woman with a part-solid nodule in the lower lobe of the left lung. Total tumor size was 2.0 cm and solid size was 1.3 cm. (**b**, **c**) Axial and a maximum intensity projection image on FDG-PET/CT shows that FDG uptake was increased slightly; the corresponding SUVmax and Deauville score were 1.32 and 2, respectively. (**d**, **e**) Histological subtype was an MIA. The pathological total tumor size was 2.0 cm, and the invasive size was 0.4 cm

**Fig. 2:**
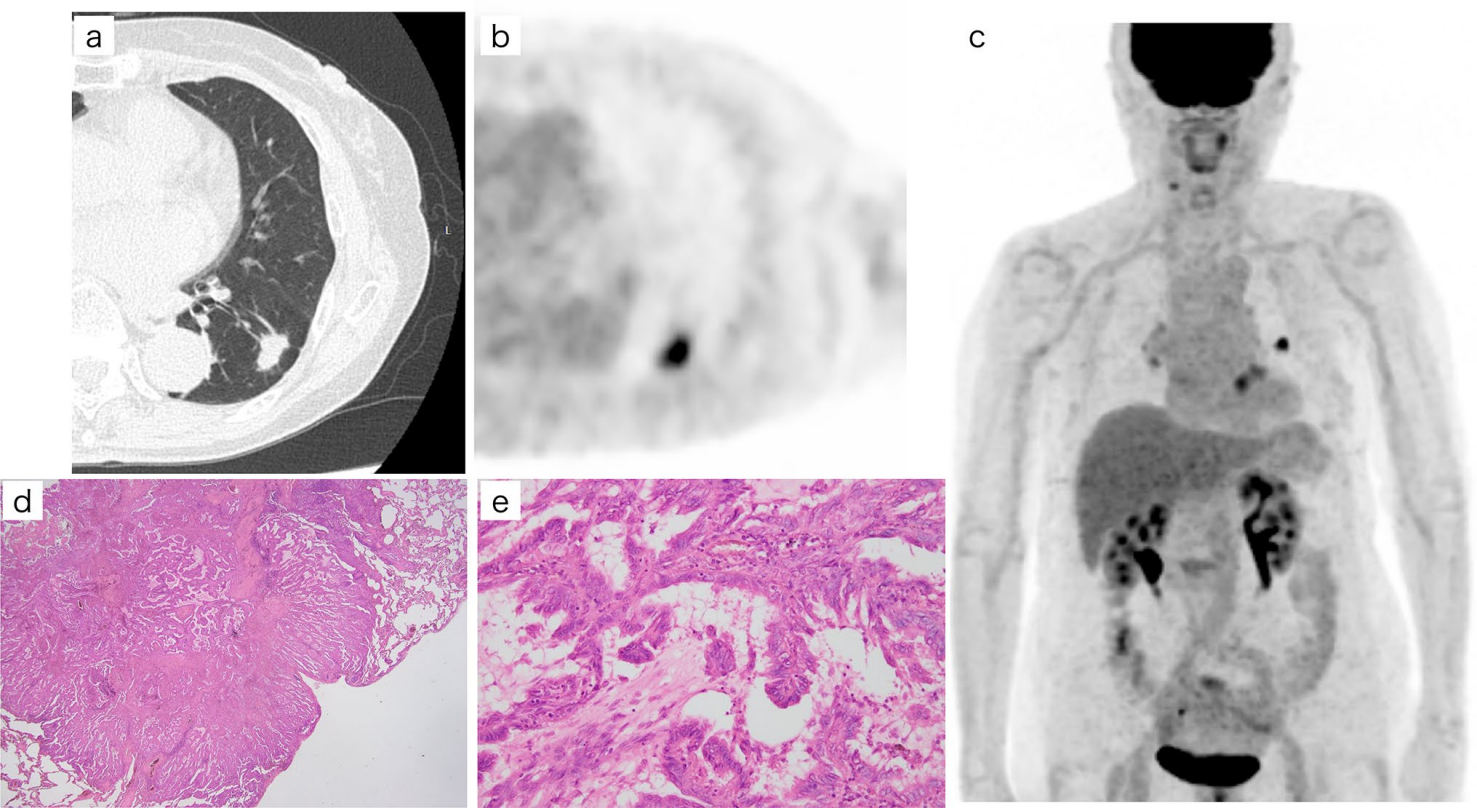
FDG-PET-CT and pathological images of a patient with invasive lung adenocarcinoma. **a** CT shows that in an 82-year-old woman with a solid nodule in the lower lobe of the left lung that the solid size was 1.6 cm. **b**, **c** Axial and a maximum intensity projection image on FDG-PET/CT shows that FDG uptake was increased strongly and the corresponding SUVmax and Deauville score were 6.51 and 5, respectively. (**d**, **e**) Histological subtype was papillary predominant lung adenocarcinoma. The pathological total and invasive size were both 1.2 cm

Fifty-one patients who had undergone complete resection for pulmonary adenocarcinoma were study participants. Lobectomy was performed for 36 lesions and a limited resection for 15 lesions. Table 1 summarizes patients’ characteristics. According to the pathologic diagnosis, of the 51 pulmonary nodules 6 were classified as less invasive (2 AIS and 4 MIA) and 45 as invasive adenocarcinoma. The predominant subtype diagnosis was available for all those in the invasive adenocarcinoma group. Figures 1 and 2 show typical PET/CT images from the less invasive and invasive adenocarcinoma groups, respectively.

**Table 1:** Clinical characterises of study patients

Characteristics	Tumor	
	Less invasive (*n*=6)	Invasive (*n*=45)
Age (years)		
Median	67	76
Range	46–78	56–86
Sex		
Men	1	20
Women	5	25
Smoking history		
Yes	2	24
No	4	21
Pathological T stage		
Tis	2	0
T1mi	4	0
T1a	0	6
T1b	0	19
T1c	0	12
T2a	0	8
Histological subtype		
Adenocarcinoma in situ	2	0
Minimally invasive adenocarcinoma	4	0
Lepidic predominant	0	3
Papillary predominant	0	32
Acinar predominant	0	6
Solid predominant	0	4

### SUVmax, total lesion size on CT, and solid size on CT of lung adenocarcinoma

The median SUVmax, total size on CT, and solid size on CT for the 51 pulmonary adenocarcinomas were 3.75 (range, 0.76–16.50), 2.4 cm (range, 0.8–4.7 cm), and 1.9 cm (range, 0–4.7 cm), respectively. The median SUVmax values for AIS, MIA, and invasive adenocarcinoma were 1.11 (range, 0.87–1.35), 1.38 (range, 0.89–3.75), and 4.97 (range, 0.76–16.50), respectively. Median total sizes on CT of AIS, MIA, and invasive adenocarcinoma were 1.4 cm (range, 1.3–1.5), 2.5 cm (range, 1–2.7), and 2.4 cm (range, 0.8–4.7), respectively. The median solid sizes for AIS, MIA, and invasive adenocarcinoma on CT were 0.25 cm (range, 0–0.5), 1.3 cm (range, 0.3–2.6), and 1.95 cm (range, 0.7–4.7), respectively.

### Deauville scores for lung adenocarcinoma

Of the six patients with less invasive adenocarcinoma the Deauville score was 1 in three patients and 2 in three patients. As to the 45 invasive adenocarcinomas the Deauville score was 1 in 1 patient, 2 in 10 patients, 3 in 6 patients, 4 in 6 patients, and 5 in 22 patients. Deauville score 1 or 2 for invasive adenocarcinoma was classified as pathological grade 1 in 1/3 patients (33%) and pathological grade 2 in 10/38 patients (26%). No invasive adenocarcinoma with a Deauville score of 1 or 2 was classified as pathological grade 3 (0%, 0/4 patients).

### Correlation of SUVmax and Deauville scores

The median SUVmax was 0.88 (range, 0.76–1.35) for Deauville score 1, 1.75 (range, 1.14–2.44) for Deauville score 2, 3.38 (range, 2.48–3.71) for Deauville score 3, 4.02 (range, 2.8–8.54) for Deauville score 4 and 9.26 (range, 4.62–16.5) for Deauville score 5. A high correlation coefficient (*r* = 0.93) was found between the SUVmax and the Deauville score.

### Inter-rater and intra-rater agreements with Deauville score

Tables 2 and 3 show inter-rater and intra-rater agreements on Deauville scores. According to quadratic-weighted Kappa statistics, the inter-rater κ-value (rater 1–1 [first-time examination] vs. rater 2) of *κ* = 0.93 and intra-rater (rater 1–1 vs. rater 1–2 [second-time examination]) k-value of *κ* = 0.97 for Deauville scores indicated high agreement.

**Table 2:** Inter-rater agreement for Deauville scores

		Rater 1-1					
		Score 1	Score 2	Score 3	Score 4	Score 5	Total
Rater 2	Score 1	4	5	0	0	0	9
	Score 2	0	8	3	1	0	12
	Score 3	0	0	3	3	0	6
	Score 4	0	0	0	1	2	3
	Score 5	0	0	0	1	20	21
	Total	4	13	6	6	22	51

**Table 3:** Intra-rater agreement for Deauville scores

		Rater 1-1					
		Score 1	Score 2	Score 3	Score 4	Score 5	Total
Rater 1-2	Score 1	3	0	0	0	0	3
	Score 2	1	13	1	0	0	15
	Score 3	0	0	5	2	0	7
	Score 4	0	0	0	3	1	4
	Score 5	0	0	0	1	21	22
	Total	4	13	6	6	22	51

### Differentiation of lung invasiveness using image-based analysis

Table 4 summarizes the differentiation of lung invasiveness using image-based analysis. There was a significant difference in CT solid size (*p* < 0.01), Deauville score for both rater 1 (*p* < 0.01) and rater 2 (*p* < 0.01), and SUVmax (*p* < 0.01) between the less invasive and invasive adenocarcinoma groups. On the other hand, there was no significant difference in CT total size (*p* = 0.21) between the two groups.

**Table 4:** Differentiation of lung invasiveness using Image-based analysis

Tumor			
	Less invasive (*n*= 6)	Invasive (*n*= 6)	*p* value
CT : Total size (cm)	1.75(1–2.7)	2.4(0.8–4.7)	0.21
CT : Solid size (cm)	0.65(0–2.5)	2(0.7–4.7)	<0.01*
PET : Deauville score of rater 1	1.5(1–2)	4(1–5)	<0.01*
PET : Deauville score of rater 2	1(1–2)	4(1–5)	<0.01*
PET : SUVmax	1.34(0.87–2.39)	4.73(0.76–16.5)	<0.01*

SUVmax maximum standardized uptake value

“Deauville score of rater 1” indicates the first time rater 1 performed the examination

*p value < 0.05 indicates a statistically significant difference

### ROC

**Fig. 3:**
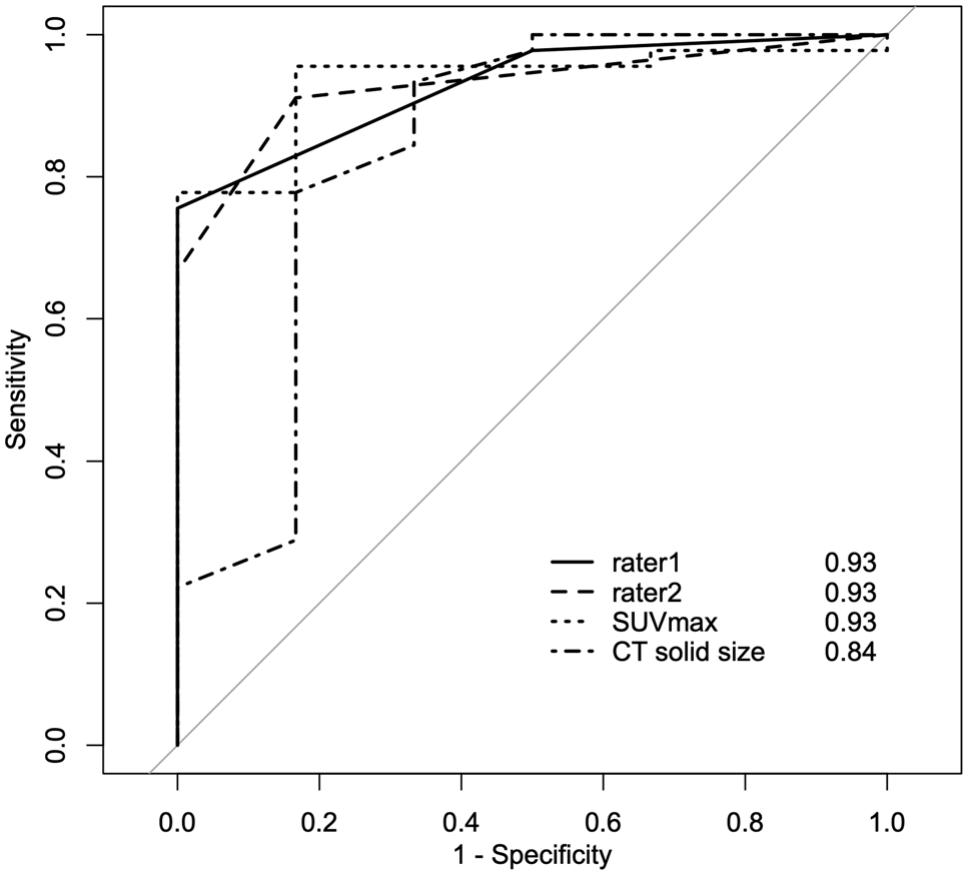
Receiver operating characteristic (ROC) curve for the Deauville score (rater 1 at the first examination and rater 2), SUVmax, and CT solid size

Table 5 summarizes the AUCs from ROC analysis of the Deauville score, SUVmax, and CT solid size. Figure 3 depicts ROC curves for each method. ROCs for the Deauville score (AUC = 0.93 for both rater 1 and rater 2) and SUVmax (AUC = 0.93) showed equivalent accuracy. The AUC for CT solid size was slightly lower (AUC = 0.84) than visual and semiquantitative FDG-PET/CT assessments. However, there were no significant differences between CT solid size and FDG-PET/CT assessments (CT solid size vs. Deauville score by rater 1, *p* = 0.41; Deauville score by rater 2, *p* = 0.49; and SUVmax, *p* = 0.46).

**Table 5:** Receiver operating characteristic curve analysis

	ROC				
	AUC	95%CI	Cut off value	Sensitivity (%)	Specificity (%)
Deauville score: rater1	0.93	0.86–1	2.5	75.60	100.0
Deauville score: rater2	0.93	0.86–1	1.5	91.10	83.3
SUVmax	0.93	0.85–1	1.45	95.60	83.30
CT solid size	0.84	0.60–1	1.35	77.80	83.30

SUVmax maximum standardized uptake value

“Deauville score: rater 1” indicates the first time rater 1 performed the examination

## Discussion

Because invasive and less invasive adenocarcinoma (AIS and MIA) can potentially be treated differently, it is crucial for surgeons to preoperatively estimate a tumor's invasiveness to determine the appropriate treatment. However, pathologic findings cannot be obtained preoperatively. Therefore, prediction of the presence of a less invasive adenocarcinoma through preoperative imaging is important for clinical decision-making when treating patients with early-stage lung adenocarcinoma. It has been reported that total size and solid component size that can be derived from CT imaging can be used to differentiate between invasive and less invasive lung adenocarcinoma [9, 18]. However, because CT imaging provides only a morphological evaluation, the potential for differentiation of invasiveness by size alone remains debatable. For a more accurate preoperative diagnosis, we must incorporate different types of imaging modalities.

In the present study, we clarified that the Deauville score was an aid in predicting pathological invasiveness of early lung adenocarcinoma. According to rater 1–1, all less invasive adenocarcinomas had a Deauville score of 1 or 2. No patient with pathological Grade 3 invasive adenocarcinoma was given a Deauville score of 1 or 2. Previously, Kagimoto et al. reported that the Deauville score was useful in predicting not only lymph node metastasis but also lymphatic, vascular, or visceral pleural invasion of early-stage lung adenocarcinoma [17]. Our study demonstrated that the Deauville score can also be applied to predict less invasive lung adenocarcinomas, including AIS and MIA. This indicates that the Deauville score has the potential to reflect the degree of pathological malignancy of a tumor. FDG-PET/CT is an important imaging tool for evaluating primary lung cancer prior to surgery and provides a number of semiquantitative parameters, with SUVmax being the most commonly used [19, 20]. It was noted that the preoperative evaluation of SUVmax could help to distinguish pathological invasiveness of lung adenocarcinoma [8]. However, access is needed to a workstation suitable for PET/CT imaging to assess SUVmax. Also, SUVmax is variable among studies and institutions according to differences in imaging protocols, equipment, etc. so it cannot be used as inclusion criteria. Assessment using Deauville criteria based on FDG-PET/CT findings can eliminate those disadvantages of SUVmax [14]. Our present results showed a high correlation coefficient between SUVmax and Deauville scores. The AUCs of Deauville scores were comparable to SUVmax. Moreover, each Deauville score covered a large area expressed as an SUVmax. Using Deauville criteria, we can assess the degree of FDG uptake succinctly and efficiently rather than using SUVmax.

We found no significant differences between the AUCs of the Deauville score and CT solid size. That the preoperative solid size on CT can help differentiate AIS and MIA from invasive adenocarcinomas was reported [21]. Our study indicated that Deauville scores achieved good diagnosability, similar to CT solid size. Furthermore, there were no significant differences between CT solid size and FDG-PET/CT assessments but the Deauville score and SUVmax had slightly higher AUCs in our study than CT solid size for predicting the invasiveness of lung adenocarcinoma. The AUC of CT solid size (0.84) was a little lower than the lower limit of the 95% confidence interval (CI) for the Deauville score (95%CI 0.86–1) and SUVmax (95%CI 0.85–1). Solid size measurement involves issues of inconsistency with the invasive size determined using pathological specimens [22]. The solid size shown on thin-section CT may be larger than that of the actual invasive component, because the solid components shown on CT contain cancer cells as well as alveolar collapse, fibrosis, etc. Although PET/CT has lower spatial resolution than CT, the degree of FDG accumulation more accurately reflects tumor invasiveness [23]. Therefore, using the Deauville score based on FDG-PET/CT is an attempt to resolve these issues related to CT. However, due to the possibility of sample bias and poor statistical power the consequences of using these results are difficult to determine. Further validation is needed to clarify differences in the diagnostic value between the Deauville score and CT findings. The combination of FDG-PET/CT and CT is widely used for imaging protocols before surgery. At present, thoracic surgeons mainly use CT imaging to determine indications for limited resection of early lung adenocarcinoma. The results of the present study indicate that when deciding upon the operative method, the Deauville score should be considered.

In a recent study, Choi et al. reported a high degree of interobserver agreement for the measurement of solid components of lung adenocarcinoma between a radiologist and pulmonologists and between residents versus fellows [24]. Furthermore, excellent agreement between raters for Hodgkin lymphoma was reported for the Deauville score [14].To our knowledge, the present study is the first to examine whether the criteria for the Deauville score are robust enough to permit good reliability for assessing early lung adenocarcinoma. The results showed that it was sufficiently robust for that purpose. Furthermore, it was notable that after a short period of instruction using a published manuscript describing the Deauville score, a medical student without PET experience could use the Deauville score. Deauville score is a simple and dependable visual measurement for evaluating the pathological invasiveness of lung adenocarcinoma on PET. This method simply requires the use of the eyes and some fundamental knowledge of FDG-PET/CT. We anticipate that the Deauville score may be valuable for thoracic surgeons and other experts who do not specialize in PET diagnostics, not only radiologists.

Our study had some limitations. First, this was a retrospective study and involved data from a single institution. As a result, there is a possibility of bias. Second, the sample size was relatively small, especially the number of less invasive adenocarcinoma cases. Therefore, further studies with a larger patient population are required to confirm our results. However, this is the first report to evaluate the reliability and validity of the Deauville score for predicting pathological invasiveness of early lung carcinoma. Based on the present results, it appears that the Deauville score for lung cancer will be a critical consideration by surgeons as part of preoperative examinations for lung cancer.

In conclusion, with FDG-PET/CT a visual assessment using the Deauville score correlated with pathological invasiveness of early lung adenocarcinoma. The Deauville score is a simple and dependable visual measure that might assist thoracic surgeons in planning minimally invasive surgery for early lung adenocarcinoma.
